# Reviewer acknowledgement 2013

**DOI:** 10.1186/1471-2288-14-12

**Published:** 2014-02-03

**Authors:** Irene Pala

**Affiliations:** 1 BioMed Central, 236 Gray’s Inn Road, London WC1X 8HB, UK

## Abstract

**Contributing reviewers:**

The editors of BMC Medical Research Methodology would like to thank all our reviewers who have contributed their time to the journal in Volume 13 (2013).

## 

Taghreed Adam

Switzerland

Noori Akhtar-Danesh

Canada

Arthur Allignol

Germany

Rhoda Allison

UK

Heather Allore

USA

Piero Almasio

Italy

Philip Anderson

Australia

Rebecca Andridge

USA

Cono Ariti

USA

Suzanne Audrey

UK

Matthias Augustin

Germany

Juan J Aurrekoetxea

Spain

Teresa Bago d'Uva

Netherlands

Karen Barker

UK

Betsy Jane Becker

USA

Wendy Bedwell

USA

Melanie Bell

USA

Jason Beneciuk

USA

Jason Bentley

Australia

Leslie Bernstein

USA

Ricardo V Bessa-Nogueira

Brazil

Kirsten Beyer

USA

Andreas Beyerlein

Germany

Michela Bia

Luxembourg

Harald Binder

Germany

Ingrid Binswanger

USA

Gallus Bischof

Germany

Martin Bland

UK

Emily Blood

USA

Alex Bottle

UK

Isabelle Boutron

France

Anna Brettschneider

Germany

Kyle Brothers

USA

Chad Brown

USA

Maarten Buis

Germany

Helen Burchett

UK

Alex Burdorf

Netherlands

Igor Burstyn

USA

Richard Burt

USA

Kunthel By

USA

Bo Cai

USA

Anyela Camargo

UK

Math Candel

Netherlands

Josep Carrasco

Spain

Fran Carroll

UK

Bendix Carstensen

Denmark

Enrique Casalino

France

John Castelloe

USA

Santanu Chakraborty

USA

Cole Chapman

USA

Sarah Chapman

UK

Bingshu Chen

Canada

Chyong-Mei Chen

Taiwan

Dung-Tsa Chen

USA

Guanmin Chen

Canada

Maggie Chen

Canada

Nai-Wei Chen

USA

Shuo Chen

USA

Yuh-Ing Chen

Taiwan

Zhengjia Chen

USA

Zhuo Chen

USA

Andrea Cherrington

UK

ken cheung

USA

Carolyn Chew-Graham

UK

Yasutaka Chiba

Japan

Jacqueline Rose Chinkonde

Norway

Young Cho

USA

Seung Choi

USA

Kaj Sparle Christensen

Denmark

Rong Chu

Canada

David Clark

USA

Rachel Colley

Canada

Gary Collins

UK

Christopher Colvin

South Africa

Warren Comulada

USA

Nancy Cook

USA

Catherine Crespi

USA

David Cromwell

UK

Kate Curtis

Australia

Anna Dahl

Sweden

Gavin Daker-White

UK

Raechel Damarell

Australia

Qianyu Dang

USA

Sandra De Groote

USA

Thomas Debray

Netherlands

Cinzia Del Giovane

Italy

Barbara Dennison

USA

Vikrant Deshmukh

USA

Claus Dethlefsen

Denmark

Shayesta Dhalla

Canada

Fredi Alexander Diaz-Quijano

Colombia

Markus K Diener

Germany

Rositsa B Dimova

USA

Mary Dixon-Woods

UK

Philipp Doebler

Germany

Therese Dolecek

USA

Robine Donken

Netherlands

Michael Donohue

USA

Peter Doshi

USA

Thomas Drivsholm

Denmark

Graham Dunn

UK

Geir Egil Eide

Norway

Solvig Ekblad

Sweden

Sandra Eldridge

UK

Rolf Peter Ellegast

Germany

PB English

USA

Stephen Evans

UK

Morten Wang Fagerland

Norway

Diane Fairclough

USA

Sue Fawcus

South Africa

Brian Feldman

Canada

Lambert Felix

UK

Jonathan Finks

USA

Livio Finos

Italy

Brian Fisher

USA

Sharon E Fleming

USA

Dean Follmann

USA

Edward Fottrell

UK

Matthew Fox

USA

Nick Freemantle

UK

David French

UK

Lin Fritschi

Australia

Julia Frost

UK

Halina Frydman

USA

James Gallagher

UK

Patricia Ganz

USA

Jingjing Gao

USA

Jesus M Garcia Calleja

Switzerland

Scott Garrison

Canada

Omar Carlo Gioacchino Gelo

Italy

Sebastian George

India

Thomas Gerds

Denmark

Daniel Gillen

USA

Dean Giustini

Canada

Julie Glanville

UK

Patrick Grahaam

New Zealand

Aileen Grant

UK

Maria Grant

UK

Joan Griffin

USA

Rolf Groenwold

Netherlands

Catharina Groothuis-Oudshoorn

Netherlands

Martin Gulliford

UK

Chao-Yu Guo

Taiwan

Gregory Gurevich

Israel

Lyle Gurrin

Australia

Kurinchi Gurusamy

UK

Paul Gustafson

Canada

Muhammad Hadi

UK

Michael Halasy

USA

Alix Hall

Australia

Jesper Hallas

Denmark

Taye Hamza

USA

David Hanauer

USA

Ron Leonardus Hubertus Handels

Netherlands

Sebastien Haneuse

UK

Sebastien Haneuse

USA

Peter Hannan

USA

Karin Hannes

Belgium

Frank Harrell

USA

Yulei He

USA

Larry Hearld

USA

Dick Heasman

UK

Glenn Heller

USA

Karla Hemming

UK

Kristien Hens

Netherlands

Moonseong Heo

USA

Lin Yee Hin

Australia

Mandy Ho

Australia

James Hodgkinson

UK

Michel Hof

Netherlands

Scott Hofer

Canada

Julie Horrocks

Canada

Tanya Horsley

Canada

Mike Horton

UK

Jeremy Howick

UK

Asbjørn Hróbjartsson

Denmark

Bo Hu

USA

Adwoa Hughes-Morley

UK

Wen-Han Hwang

Taiwan

Hiroo Ide

Japan

Ozlem Ilk Dag

Turkey

Justin Jagosh

Canada

Thomas Jaki

UK

Holly Janes

USA

Ryszard Janicki

Canada

Mona Jeffreys

UK

Shuang Ji

USA

Yuan Ji

USA

Huan Jiang

Canada

Ulrich John

Germany

Peter Jongen

Netherlands

Michele Jonsson Funk

USA

Galen Joseph

USA

Joshua Josephs

USA

Brennan Kahan

UK

John Kalbfleisch

USA

Joseph Kang

USA

Juha Karvanen

Finland

Monika Kastner

Canada

Jay Kaufman

Canada

Jonathan Keith

Australia

David Kent

USA

Aaron Kesselheim

USA

Ali S Khashan

Ireland

Hiromi Kikuchi

Japan

Peter Kimani

UK

Patricia Kipnis

USA

Anne Kirchhoff

USA

Olaf Klungel

Netherlands

Guido Knapp

Germany

Mirjam Knol

Netherlands

Ruwanthi Kolamunnage Dona

UK

Paul Komesaroff

Australia

Joseph Koopmeiners

USA

David Kriebel

USA

P Paul F M Kuijer

Netherlands

Malhar Kumar

India

Yong-Fang Kuo

USA

Maarit Laaksonen

Finland

Peter Lachenbruch

USA

Sarah Lamb

UK

Paul Landais

France

Britta Lang

Germany

Peter Langhorne

UK

Klaus Langohr

Spain

Eduardo Lazcano-Ponce

Mexico

Liana Leach

Australia

Michael Lechner

Switzerland

Edith Leclercq

Netherlands

Katherine Jane Lee

Australia

Sunghee Lee

USA

Wen-Chung Lee

Taiwan

Shelley Lees

UK

Annabelle Lemenuel

Switzerland

Sam Lendle

USA

Belinda Lennox

UK

Russell Lenth

USA

Mary Lesperance

Canada

Lenard Lesser

USA

Danielle Levac

Canada

Joel Lexchin

Canada

Dalin Li

USA

Jialiang Li

Singapore

Liang Li

USA

Ruosha Li

USA

Shu Qin Li

Australia

Xuefeng Li

USA

Hester Floor Lingsma

Netherlands

Wendy Lipworth

Australia

Danping Liu

USA

Guo xiang Liu

China

Ivy Liu

New Zealand

Craig Lockwood

Australia

Jose-Antonio Lopez-Pina

Spain

Jody Lori

USA

Liang-Jing Lu

China

Jay Lubin

USA

Vittoria Lutje

UK

Kate Lyden

USA

Jinhui Ma

Canada

Jason Madan

UK

Kepher Makambi

USA

Micha Mandel

Israel

Ian Marschner

Australia

Andrea Marshall

UK

Monique Martin

UK

Ana Marusic

Croatia

Jean Yves Mary

France

John Matthews

UK

Elaine Maxwell

UK

Douglas Maynard

USA

Anthony Mbonye

Uganda

Lawrence Mbuagbaw

Cameroon

Annabel McGuire

Australia

Qingyue Meng

China

Joris Menten

Belgium

Lisa Merry

Canada

Bita Mesgarpour

Austria

Craig Meyer

USA

David Meyre

Canada

David Miller

USA

Frank Miller

Sweden

Edward Mills

Canada

Trifon Missov

Germany

Martina Mittlbock

Austria

Sigrun Mogedal

Norway

David Moher

Canada

Sassy Molyneux

Kenya

Matteo Monami

Italy

Victor Montori

USA

Zena Moore

Ireland

David Mosen

USA

David Murray

USA

Celine Murrin

Ireland

Jessica Myers

USA

Robert Nachbar

USA

Meredith Nahm

USA

Pengsheng Ni

USA

Pentti Nieminen

Finland

Marie Norredam

Denmark

Susan L Norris

USA

Peter Nowak

Austria

Matthias Nuebling

Germany

Owen O'Donnell

Greece

David Ohlssen

USA

Roberto S. Oliveri

Denmark

Craig Olsson

Australia

Yi Pan

USA

Gareth Parry

USA

Jacoby Patterson

UK

Robert Patton

UK

Margaret Pepe

USA

Jaime Peters

UK

Pete Philipson

UK

Olivia Phung

USA

A Simon Pickard

USA

Ruth Pickering

UK

Elizabeth Pienaar

South Africa

Carol Pierannunzi

USA

Bart G Pijls

Netherlands

Catherine Pirkle

Canada

Pierre Pluye

Canada

Gregory Pond

Canada

Alan Poots

UK

Christine Porzelius

Germany

Koen B Pouwels

Netherlands

Justin Presseau

UK

Eleanor Pullenayegum

Canada

Jing Qian

USA

Rui Qin

USA

Peihua Qiu

USA

Ethel Quayle

UK

Karen Rabin

USA

Michael Racz

USA

Sally Rankin

USA

Jeremy Rassen

USA

Sujit D Rathod

USA

David Redden

USA

David Resnik

USA

Jo Rick

UK

Jennifer Ridgeway

USA

Gerald Riley

USA

Oliver Rivero-Arias

UK

Mark Rodgers

UK

Melissa Rolfes

USA

Hela Romdhani

Canada

Ulrike Rothe

Germany

Jason Roy

USA

Gerta Rücker

Germany

Montserrat Rue

Spain

Robert Rush

UK

Margaret Ryan

USA

Sanjeeve Sabharwal

UK

Shubhayu Saha

USA

Paramita Saha Chaudhuri

USA

Georgia Salanti

UK

Georgia Salanti

Greece

Frank Samuelson

USA

Julio Sánchez-Meca

Spain

Eric Sandler

USA

Nancy Santesso

Canada

Hiroyuki Sasai

USA

Jelena Savovic

UK

Kourosh Sayehmiri

Iran

Roberta Scherer

USA

Peter Schlattmann

Germany

Kurt Schmidlin

Switzerland

David Schoenfeld

USA

Matthew Scotch

USA

Richard Scranton

USA

Kate Seers

UK

Stephen Senn

Luxembourg

Shulian Shang

USA

Mona Sharifi

USA

Stephen Sharp

UK

Michele Shedlin

USA

Ian Shemilt

UK

Richard Shewchuk

USA

Mohamed Shoukri

Saudi Arabia

Mohamed shoukri

Canada

Julius Sim

UK

Siva Sivaganesan

USA

Mitchell Smith

Australia

Niels Smits

Netherlands

Janet Smylie

Canada

Kym Snell

UK

Marta Soares

UK

Fujian Song

UK

William Sorensen

USA

Donna Spiegelman

USA

Cathie Spino

USA

Sheila Sprague

Canada

Andreas Stang

Germany

Ewout Steyerberg

Netherlands

Manfred Stommel

USA

Matthew Strickland

USA

Koji Sugiyama

Japan

Gerhard Sulo

Norway

Lei Sun

Canada

Josefin Sundin

UK

Alex Sutton

UK

Michael Szarek

USA

Adam Szpiro

USA

Ziad Taib

Sweden

Monica Taljaard

Canada

Larry Tang

USA

Niansheng Tang

China

Armando Teixeira-Pinto

Australia

Hla-Hla Thein

Canada

Markus Themessl-Huber

UK

Denise Thew

USA

James Thomas

UK

Kamala Thriemer

Belgium

Hans Thulesius

Sweden

Justin Timbie

USA

Fiona Timmins

Ireland

Brian Timmons

Canada

Diana Tisnado

USA

Andrew Titman

UK

Allison Tong

Australia

Tom Trikalinos

USA

Miao-Yu Tsai

Taiwan

Dongsheng Tu

Canada

Reginald Tucker-Seeley

USA

Deborah Turnbull

Australia

Lucy Turner

Canada

Selda Ulucanlar

UK

Anneli Uuskula

Estonia

Linda Valeri

USA

Peter van der heijden

Netherlands

Marjon van der Pol

UK

Jenny Theodora van der Steen

Netherlands

Dwayne Van Eerd

Canada

Emily Van Meter

USA

Michele Vecchione

Italy

Jan Verbakel

Belgium

Hilde Verbeek

Netherlands

Jay Verkuilen

USA

Andrew Vickers

USA

Wolfgang Viechtbauer

Netherlands

Paolo Villari

Italy

Natale Vincenzo

Italy

André O von Bueren

Germany

Bert Vrijhoef

Singapore

Sholom Wacholder

USA

Willem Waegeman

Belgium

Ming Wang

USA

Yi Wang

USA

Zhen Wang

USA

Christopher Weir

UK

Mary Wells

UK

Sijin Wen

USA

Helene Werner

Switzerland

Peter Westfall

USA

Philip Westgate

USA

Ian R White

UK

Matthew White

USA

Penny Whiting

UK

Andreas Wienke

Germany

Adam Wilcox

USA

Brian Willis

UK

Val Wilson

Australia

Luke Wolfenden

Australia

Eliza Wong

Hong Kong

Geoff Wong

UK

Qing Wu

USA

Sheng Wu

USA

Yukun Wu

USA

Stefan Wuchty

USA

Lingzhong Xu

China

Xiaonan Xue

USA

Hsin-Chou Yang

Taiwan

Su-Fen Yang

Taiwan

Zhao Yang

USA

Barbara Yawn

USA

Reza Yousefi-Nooraie

Canada

Jihnhee Yu

USA

Recai Yucel

USA

Antonia Zapf

Germany

Philip Zazove

USA

Bo Zhang

USA

Jing Zhang

USA

Nanhua Zhang

USA

Song Zhang

USA

Xiaoxi Zhang

USA

Xu Zhang

USA

Zheng Zhang

USA

Ling Zhao

China

Hui Zheng

USA

Wei Zhong

USA

Xiaofeng Zhou

USA

Ming Zhu

USA

Jeanette Ziegenfuss

USA

Corwin Zigler

USA

Jörg Zinserling

Germany

Merrick Zwarenstein

Canada

## Pre-publication history

The pre-publication history for this paper can be accessed here:

http://www.biomedcentral.com/1471-2288/14/12/prepub

